# Modeling the Impact of Newcastle Disease Virus Vaccinations on Chicken Production Systems in Northeastern Madagascar

**DOI:** 10.3389/fvets.2019.00305

**Published:** 2019-09-26

**Authors:** Akshaya Annapragada, Cortni Borgerson, Sarah Iams, M. Ando Ravelomanantsoa, Graham C. Crawford, Marika Helin, Evelin Jean Gasta Anjaranirina, Hervet J. Randriamady, Christopher D. Golden

**Affiliations:** ^1^Harvard John A. Paulson School of Engineering and Applied Sciences, Harvard University, Cambridge, MA, United States; ^2^Department of Anthropology, Montclair State University, Montclair, NJ, United States; ^3^Madagascar Health and Environmental Research (MAHERY), Maroantsetra, Madagascar; ^4^Departement Production et Partenariat, Institut Malgache des Vaccins Vétérinaires (IMVAVET), Ampandrianomby, Antananarivo, Madagascar; ^5^Wildlife Health Network, Sonoma, CA, United States; ^6^Department of Nutrition, Harvard TH Chan School of Public Health, Boston, MA, United States

**Keywords:** poultry, vaccination, food security, malnutrition, animal-source foods, sustainable agriculture

## Abstract

Chickens are a key source of nutrition for rural Malagasy communities. Due to high endemic rates of Newcastle disease, it remains challenging to raise sustainable chicken flocks as a consistent food source. Here, we explore the impact of triannual Newcastle disease virus (NDV) vaccine interventions on the growth and herd immunity acquisition of Malagasy chicken flocks. Between 2011 and 2018 we collected longitudinal data to assess the population dynamics of chicken populations in remote Malagasy communities. In 2016, we launched a pilot campaign for vaccination in six rural communities to determine the impacts on chicken survivorship and productivity. We used these data to specify a mathematical model of realistic Malagasy chicken population dynamics under a triannual vaccination regime. The mathematical model represents an extension to conventional SIR models that allows for modeling the impact of specific vaccinations on chicken flock dynamics, rather than estimation of parameters. Understanding chicken population dynamics is important for realizing the potential for domestic chicken flocks to serve as sustainable food sources. The results suggested that vaccination coverage of at least ~40% is necessary over 5+ years to achieve population doubling, while complete herd immunity may not be possible given the short duration of effectiveness of vaccination, and the high levels of births and deaths in the chicken flocks.

## 1. Introduction

One of many challenges faced by remote Malagasy communities is achieving food security (1). For millennia, Malagasy have hunted and eaten wildlife for food, including birds, tenrecs, bats, carnivores, and lemurs (1–5). While this meat is rich in nutrients and may have historically been plentiful, wildlife stocks have steadily declined in response to overhunting and environmental changes (6–8). This scarcity is, in part, responsible for the severe malnutrition found in Malagasy communities, where close to 50% of children under 5 years old are stunted (9). A potential conservation and development solution that has been posited to wean people from wildlife without compromising nutritional status is to replace the meat of wild animals with that from chickens.

While chicken has potential to replace wildlife nutritionally, raising domestic chicken flocks in remote, rural communities in Madagascar is a complex endeavor. Primarily, chicken has not served as a consistent and economical source of food in many parts of Madagascar because community poultry flocks are vulnerable to Newcastle disease (10–12). Newcastle disease is a virus that causes neurological, respiratory, and gastrointestinal symptoms in poultry (13), and is often fatal, hence preventing the stable establishment of productive chicken flocks. Newcastle disease is endemic across most of Africa, including Madagascar (14, 15), and estimates suggest that Newcastle disease outbreaks can result in mortality from 50 to 100% of a chicken flock (11). Moreover, mathematical modeling suggests that poultry diseases, including Newcastle disease, can result in a loss of income between 10 and 25% among poor, rural populations in Madagascar (16). For this reason, mitigating the negative impacts of Newcastle disease on chicken flocks in Madagascar is a necessary step towards improving food security (10, 11, 17) and enabling the replacement of wildlife consumption.

In regions where Newcastle disease poses an obstacle to raising chickens, vaccine interventions have the potential to drastically increase productivity, hence making chickens more viable for consumption and raising (11, 18). Vaccination campaigns for the control of Newcastle disease further have the potential to stabilize chicken flocks and may facilitate an increase in the sale of chicken to peri-urban or urban areas, generating new income that can be channeled to support food security (10). In particular, successful Newcastle disease virus vaccination campaigns in Australia have led to control of Newcastle disease there and have been expanded to countries across Africa and Asia (11). Field trials of the Newcastle disease vaccination interventions in Tanzania, Myanmar, and South Africa have demonstrated that vaccination interventions can increase chicken immunity, chicken survival, chicken and egg consumption, and chicken flock size (19–22).

Despite these encouraging results, poultry vaccination campaigns in remote regions of Madagascar pose significant challenges, such as the maintenance of a cold chain, recruiting trained professionals to administer the vaccines, and encouraging community participation. With regard to these challenges, the I-2 ND vaccination against Newcastle disease offers a potential solution. The I-2 ND vaccine is especially suitable for use in rural environments because it is thermo-tolerant, does not require refrigeration, and can be administered by eye-dropper rather than injection (23). In field trials in Western Amhara, Ethiopia, I-2 ND vaccination campaigns reduced chicken mortality by 82%, and in Mozambique, chickens vaccinated with the I-2 ND vaccine were shown to be 5 times less likely to die than unvaccinated chickens (24, 25). However, the vaccine is effective only for four months, hence requiring triannual administration (23). It is an open question whether stable and growing chicken populations with herd immunity can be achieved in Madagascar through use of the I-2 ND vaccine.

While existing epidemiological models of Newcastle disease using the conventional R0/SIR framework effectively model the spread of Newcastle disease, they do not evaluate the effect of specific vaccination regimes and coverage rates on immunity acquisition and population stability and growth (26). In particular, Van Boven et al. modeled Newcastle disease using an SEIR compartmental model to characterize the spread of Newcastle disease and estimate the R parameter that defines the reproduction number of Newcastle disease virus (26). This model used data from experiments conducted in flocks of broiler chickens to specify the model and concluded that herd immunity from Newcastle disease would require >85% of chickens in a flock to have immunity at all times.

However, this class of epidemiological model does not quantify the vaccination coverage levels needed to achieve this herd immunity threshold and population growth in a population subject to realistic inflow and outflow dynamics. Moreover, it does not incorporate the need for triannual vaccinations, an important constraint on the use of the I-2 ND vaccine. Therefore, we constructed a mathematical model of chicken population dynamics, including birth rates, death rates, sale rates, purchase rates, and consumption rates, under a triannual vaccination regime. We specified the model using parameter estimation from field survey data of chicken flocks in nine Malagasy communities. Chicken flocks in Madagascar are of East African ancestry (27), and consist of domestic, village chickens (not layers, hybrids, or other commercial chickens). The model was used to estimate the levels of triannual vaccination coverage required to maintain the herd immunity threshold, and to stabilize the population and enable growth. Finally, a poultry vaccination program was initiated in six remote communities in order to evaluate the potential to achieve these vaccination levels in such communities.

In order to improve the nutritional status of local people and protect the surrounding wildlife populations from wildlife hunting, it is necessary to specify and deploy effective interventions to lessen the burden of Newcastle disease. Therefore, evaluating the vaccination coverage necessary to mitigate the impacts of Newcastle disease, and establishing a program in Madagascar is a necessary step towards establishing chicken as a reliable food source. More broadly, this modeling framework is applicable to studies of how specific vaccination regimes impact population growth and herd immunity under realistic population dynamics.

## 2. Methods

### 2.1. Longitudinal Survey

The Madagascar Health and Environmental Research (MAHERY) team collected longitudinal data from 2011–2018 in nine rural Malagasy communities throughout the Makira and Masoala rainforests of northeastern Madagascar (Table 1). Surveys varied, and included questions on household-level chicken ownership (9 of 9 communities), demographics and survivorship (9 of 9 communities), egg laying (6 of 9 communities) and hatching (6 of 9 communities), consumption (2 of 9 communities), sales (3 of 9 communities), deaths (due to illness, and other causes) (6 of 9 communities), and vaccination (6 of 9 communities).

**Table 1:** A tabular representation of where, when and what data was utilized.

**Community**	**Parameter**						
	**Consumption**	**Deaths: illness**	**Deathes: theft and predation**	**Purchases**	**Hatches**	**Sales**	**Vaccination**
1	⊲	°	°	°	°⊳		†
2		°	°	°	°⊳		
3		°	°	°	°⊳		
4		°*	°*	°*	°*⊳**		†
5	⊲	°*	°*	°*	°*⊳**		
6		°*	°*	°*	°*⊳**		†
7						‡	†***
8						‡	†***
9						‡	†***
Total chicken owning samples	N/A	3,326	3,326	3,326	3,303	295	

⊲ *Dataset 1: Dietary composition data = Annual 2013–2014*.

° *Dataset 2: Ownership = Monthly, August 2011–November 2013, February 2014–January 2017*.

°* *Monthly September 2015–January 2017*.

⊳ *Dataset 3: Hens = Monthly August 2011–December 2013, February 2014–April 2016*.

⊳** *Monthly September 2015–January 2017*.

‡ *Dataset 4: Ownership and transactions = Every 4 months, April 2016–January 2017*.

† *Dataset 5: Vaccination = Every 4 months, May 2016–September 2018*.

†*** *Every 4 months, May 2016–January 2018*.

### 2.2. Vaccination Intervention

Beginning in May 2016, a vaccination intervention was initiated in six of the nine communities. The I-2 ND vaccine is produced by the Malagasy Institute for Veterinary Vaccines, IMVAVET (Institut Malgache des Vaccins Vétérinaires) in the capital city of Antananarivo. Prior to each tri-annual vaccination campaign, vaccines are couriered by air in a cold box (maintained at 4° C) to the peri-urban center of Maroantsetra where master vaccinators deliver the vaccine by motorcycle, boat, and foot, to the pilot communities. Keeping the thermotolerant vaccine refrigerated for as long as possible extends its efficacy. Once taken out of the 4°C cold box at the peri-urban center, the vaccine is kept as cool as possible by keeping it out of the sun and nestled in a damp cotton cloth within a loose weave basket during its distribution to remote communities (28). In order to confer immunity to the flocks prior to known seasonal peak periods of Newcastle disease outbreaks, each of the three tri-annual vaccine campaigns are designed to vaccinate chickens about one month before each outbreak. In the community, a trained para-vet who is a member of the community carries out door-to-door vaccination over several days. This community vaccinator purchases the vaccine vials and charges a fee for each dose, keeping the small net as compensation for their efforts. The vaccine is manufactured to order; therefore, community vaccinators take orders during vaccinations for the subsequent campaign. This structure of relying on trained community vaccinators, under the management of master vaccinators in the population centers, allows for a connection between the remote regions taking part in the vaccination campaign, and the population centers where the vaccine is manufactured.

### 2.3. Analysis of Vaccination Coverage

We analyzed the longitudinal vaccination data to determine levels of vaccination coverage currently present in each community. For communities 7, 8, and 9, all chickens in the community were surveyed. For communities 1, 4, and 6, all vaccinated chickens were recorded in the surveys, but not all unvaccinated chickens were recorded. Since the sample was biased toward vaccinated chickens, calculating a vaccination coverage from only the given data would lead to an artificially high coverage. Therefore, we used random surveys of households to estimate the total number of chickens present in the community in June 2018. We used this to adjust all previous vaccination coverages to account for unvaccinated chickens. We accomplished this adjustment by calculating the ratio of adjusted:unadjusted coverage for June 2018, and using this as a normalization constant for coverage at previous time point where it was not possible to estimate the total number of chickens present. Therefore, the community level vaccination coverage represents the true population coverage for each community, calculated as the proportion of vaccinated chickens out of all chickens in the community, at each vaccination time point.

For the calculation of vaccination coverage, we included families that were not home during the vaccination campaign at a particular time point, but owned chickens, since their “0%” vaccination affects the flock's overall immunity. Because families moved in and out of each community during the study, there is a slight variation in the number of households in each community at each time point. We used the percentage of chickens vaccinated in each community to monitor the present progress of the vaccination campaigns, and to determine whether current vaccination coverage is on track to achieve population doubling and herd immunity, as predicted by the model.

### 2.4. Parameter Extraction

We examined changes in chicken populations over time by estimating inflows and outflows of chickens in each community. We defined outflows as chicken population losses resulting from sales and deaths due to consumption, illness, and other causes (predation or theft). We defined inflows as chicken population additions resulting from purchases and the hatching of new chicks. We treated each inflow and outflow as a parameter with a fixed range, to be extracted from the longitudinal data described above.

For hatches, purchases, sales, deaths due to illness, and deaths due to other causes (predation and theft) we aggregated the longitudinal data to calculate a household level rate for all households and available timepoints for each parameter. Using these values, we next calculated the mean value and standard deviation for each parameter across households and timepoints (Table 2). For consumption, we needed an alternative approach to estimate the parameter, since households can eat chickens, even if they do not own them. Including only chicken owning households would fail to capture this share of the consumption. Therefore, we used data from the MAHERY Cohort Study on the mean percentage of meals including chicken in a community to estimate how many total chickens a community consumed in a month (29). We then estimated the total number of chickens owned in a community by random surveys conducted in June 2018 (since total ownership data did not exist for the time period of the dietary surveys at some sites). The number of chickens eaten was divided by the total number of chickens owned to obtain an estimate of consumption as a constant proportion for the two communities for which dietary data exists. We then calculated a mean and standard deviation across these two communities.

**Table 2:** Calculations and values for hatches, purchases, sales, deaths due to illness, deaths due to other causes (predation and theft), and consumption.

**Parameter description**	**Chickens**	**N (number of households or communities considered)**	**Mean value**	**SD value**
Household ownership, *o*	*X*_*o,i*_—number of chickens owned in household, *i*	–	–	–
Death due to illness, *d*	*X*_*d,i*_—number of chickens dead due to illness in household, *i*	3,326	0.437	0.752
Death due to predation and theft, *n*	*X*_*n,i*_—number of chickens dead due to predation and theft in household, *i*	3,326	0.0565	0.243
Hatch, *h*	*X*_*h,i*_—number of chickens hatched in household, *i*	3,303	0.7474	2.962
Purchase, *p*	*X*_*p,i*_—number of chickens purchased in household, *i*	3,326	0.0176	0.126
Sales, *s*	*X*_*s,i*_—number of chickens sold in household, *i*	295	0.0270	0.0781
Community ownership, *o*	*X*_*o,i*_—number of chickens owned in community, *i*	–	–	–
Consumption, *c*	*X*_*c,i*_—number of chickens consumed in community, *i*	2	0.498	0.007

*For all k in o, d, n, h, p, s, c*.

*(1) The household (o, d, n, h, p, s) or community (c) level proportions are calculated as* $H_{k,i}=\frac{X_{k,i}}{X_{o,i}}$.

*(2) The mean value is calculated from the proportions as* $\mu_{k}=\frac{\sum_{i=1}^{N}H_{k,i}}{N}$.

*(3) The standard deviation value is calculated from the proportions and mean as* $\mu_{k}=\frac{\sum_{i=1}^{N}H_{k,i}-\mu_{k}}{N-1}$.

*Calculations for hatches, purchases, sales, deaths due to illness, and deaths due to other causes (predation and theft) are calculated at the household level, while consumption is calculated at the community level*.

The standard deviations for the 6 parameters calculated from household level-data are quite high (Table 2), indicating large variability in the parameter estimates. For this reason, we solved the mathematical model described below for the mean parameter values as well as a range of values deviating by fractions of the standard deviation.

### 2.5. Mathematical Modeling

We modeled changes in chicken populations over time from a vaccination intervention. We treated the inflow and outflow parameters in the model as known constants, extracted from the longitudinal data. We treated vaccination as an unknown variable, which we modeled for varying coverage levels. Past research has shown that the I-2 ND vaccine is effective for 4 months (23). This conclusion was based on antibody titer measurements on chickens given the I-2 ND vaccine. The study tracked antibody titers in chickens at 3 sites over 4 months, and found that in 2 sites antibody titers remained protective at the end of 4 months, while in 1 site the titers had dropped substantially by the end of 4 months (23). Therefore, in our modeling, we chose a 4 month threshold as a conservative estimate for the duration of time the I-2 ND vaccine remains effective. This study also demonstrated the efficacy of the I-2 ND against the velogenic strain of NDV, which is the most virulent (30). Here, we model the most conservative scenario—If immunity persists longer than 4 months, or less virulent strains of NDV are present, flocks will outperform our predictions. Therefore, we treated chicken flocks as two distinct populations (vaccinated and unvaccinated) at each time point, and chickens were allowed to move between the vaccinated and unvaccinated populations every four months (the interval between vaccination campaigns). Chickens move from the unvaccinated to vaccinated populations due to vaccine administration, and from the vaccinated to unvaccinated populations due to a loss of immunity caused by failure to revaccinate (Equations 1, 2), where *x*(0) = 1 and *y*(0) = 0, and *x* is the proportion of vaccinated chickens, *y* is the proportion of unvaccinated chickens, and *r* is the vaccination coverage.

(1) $$\begin{array}{l} \frac{dx}{dt}=\underset{hatches}{\underset{︸}{\mu_{h}\left(x+y\right)}}-\underset{illness\;deaths}{\underset{︸}{\mu_{d}\left(x\right)}}-\underset{theft/predation\;deaths}{\underset{︸}{\mu_{n}\left(x\right)}}-\underset{sales}{\underset{︸}{\mu_{s}\left(x\right)}}\\ \;-\underset{consumption}{\underset{︸}{\mu_{c}\left(x\right)}}+\underset{purchases}{\underset{︸}{\mu_{p}\left(x\right)}}-\underset{vaccination}{\underset{︸}{rx}}+\underset{not\;revaccinated}{\underset{︸}{\left(1-r\right)y}} \end{array}$$

(2) $$\begin{array}{l} \frac{dy}{dt}=-\underset{theft/predation\;deaths}{\underset{︸}{\mu_{n}\left(y\right)}}+\underset{vaccination}{\underset{︸}{rx}}-\underset{not\;revaccinated}{\underset{︸}{\left(1-r\right)y}} \end{array}$$

We first solved the model for the mean values of each parameter. Then, to account for variability in the values of inflow and outflow parameters, we also considered extreme cases: In the “worst-case” scenarios we used parameter values incremented by fractions of a standard deviation for each outflow parameter (deaths, sales, eating) and decremented by fractions of a standard deviation for each inflow parameter (hatches, purchases). This represents a scenario where a maximum number of chickens leave the population, with minimal additions. This is less favorable than the mean case for population growth. In the “best-case” scenarios we used parameter values decremented by a fraction of a standard deviation for each outflow parameter and incremented by a fraction of a standard deviation for each inflow parameter. This represents a scenario where a minimum number of chickens leave the population, with maximal additions. This is more favorable than the mean case for population growth. Table 3 demonstrates the various scenarios considered and how this affected each parameter value.

**Table 3:** Parameter calculations for mean-case, worst-case, and best-case scenarios.

	**Parameter value**					
**Scenario**	**Deaths due to illness**	**Deaths due to predation and theft**	**Hatch**	**Purchases**	**Sales**	**Consumption**
Mean- case	μ_*d*_	μ_*n*_	μ_*h*_	μ_*p*_	μ_*s*_	μ_*e*_
Worst case	μ_*d*_ + *mσ*_*d*_	μ_*n*_ + *mσ*_*n*_	μ_*h*_ − *mσ*_*h*_	μ_*p*_ − *m* × σ_*p*_	μ_*s*_ + *mσ*_*s*_	μ_*e*_ + *mσ*_*e*_
Best case	μ_*d*_ − *mσ*_*d*_	μ_*n*_ − *mσ*_*n*_	μ_*h*_ + *mσ*_*h*_	μ_*p*_ + *m* × σ_*p*_	μ_*s*_ − *mσ*_*s*_	μ_*e*_ − *mσ*_*e*_

*We considered the following fractions, m = 0.01, 0.05, 0.1, 0.15, in evaluating each scenario. In the mean-case scenario all parameters assume their mean value. In best-case scenarios, deaths due to illness, deaths due to predation and theft, consumption, and sales are lowered by a fraction, m, of their standard deviation, while hatch and purchases are raised by the same fraction, m, of their standard deviation. In worst-case scenarios, deaths due to illness, deaths due to predation and theft,consumption, and sales are raised by a fraction, m, of their standard deviation, while hatch and purchases are lowered by the same fraction, m, of their standard deviation*.

The following assumptions were made: (1) Deaths due to illness are entirely due to Newcastle disease; (2) the vaccination is 100% effective for only 4 months and ineffective thereafter (allowing chickens to transition immediately between the vaccinated and unvaccinated populations at each vaccination campaign); (3) parameter values are constant for the duration of the model, based on the 4-month intervals over which they were calculated (this simplifying assumption ignores short term (month-to-month) seasonal variation in parameter values, but adequately represents long term (year-to-year) behavior); (4) chickens entering the population through purchase and the hatching of new chicks are always unvaccinated; (5) the vaccination interventions conducted so far have not changed the chicken populations significantly, so longitudinal data that overlaps with these initial vaccine campaigns is still included in parameter extraction; and (6) vaccinated chickens leave the population only due to non-illness deaths (predation, theft), as families are unlikely to invest in vaccination, only to eat or sell the chicken shortly after. In the Supplementary Information, we relax the assumption of 100% effective vaccination to demonstrate how increased vaccination is needed in the event the vaccination is imperfectly effective, such as if administration is imperfect or other diseases not accounted for in the model exist which suppress chicken response to the vaccine. This model simplifies a complex disease system where other diseases (e.g., pox, cholera) are not included as forms of death. Newcastle disease virus is, however, the most prevalent form of disease locally. While we do not explicitly consider deaths from other diseases due to these reasons, our parameter estimate for illness deaths likely includes some deaths due to other illnesses with similar presentations, since the estimate was made from household surveys (chicken owners were asked to identify Newcastle disease in their flocks by common symptoms such as head drooping and green feces). Moreover, our worst-case scenario in which both Newcastle disease deaths (μ_*d*_) and other deaths (μ_*n*_) are increased by 5% of a standard deviation presents a scenario in which mortality is higher than anticipated, and gives a sense for population dynamics when excess mortality is observed.

We used *Mathematica 11.0 Student Edition* to obtain numerical solutions for the changes in population size under various vaccination coverage levels. We then analyzed the solutions to understand how different vaccination coverage levels impact population growth, population doubling, and the acquisition of herd immunity.

Population doubling refers to the time at which the population reaches twice its initial size. Herd immunity refers to the time at which a sufficient proportion of a population has become immune to disease via vaccination, that outbreaks of the disease can no longer spread. This metric varies by disease, based on its transmission rate, and virulence (31). We assume that vaccination coverage of 85% of the flock is required for herd immunity against Newcastle disease, as predicted by van Boven et. al's SEIR model (26).

### 2.6. Ethics Statement

Data concerning chicken population dynamics, trade, sale, and husbandry conditions were deemed not to require an IRB by the Harvard TH Chan School of Public Health's Office of Human Research Administration's Protocol IRB13-1862. Data concerning human dietary practices have been de-identified according to our approved research protocol, including verbal informed consent for all research participants (HSPH OHRA Protocol 22826). Written informed consent was deemed culturally inappropriate in this region where many people are illiterate and signing contracts was not common practice. Records of oral consent were maintained on our study roster sheets. All consent procedures were approved by the Harvard TH Chan School of Public Health's Office of Human Research Administration (Protocol 22826).

## 3. Results

### 3.1. Population Growth

**Figure 1:**
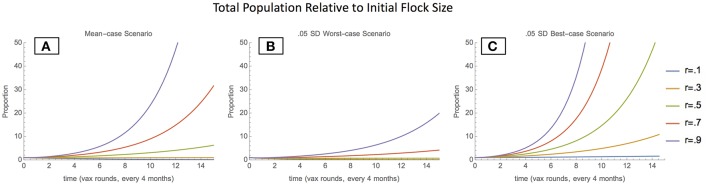
Total Population relative to initial flock size- mean-case scenario **(A)**, 0.05 SD-worst-case scenario **(B)**, and 0.05 SD-best-case scenario **(C)**.

In the mean-case scenario (mean parameter values used to solve model), the total chicken population grows with both moderate and high vaccination coverage levels, indicating that sufficient vaccination can enable population growth (Figure 1A). Scenarios around the mean-case were also considered, to demonstrate a range of possible behaviors. In the 0.05 SD worst-case scenario (inflow parameters lowered by 0.05 SD, outflow parameters raised by 0.05 SD), the total population also grows with moderate to high vaccination coverage levels; however, the total population as proportion of initial population remains much lower (Figure 1B). This indicates that in worst-case scenarios, vaccination can still enable population growth, but growth occurs far more slowly. In the 0.05 best-case scenario (inflow parameters raised by 0.05 SD, outflow parameters lowered by 0.05 SD), the total population grows rapidly even at low vaccination coverage levels, indicating that inflow and outflow conditions are favorable enough to enable exponential growth regardless of vaccination (Figure 1C).

**Figure 2:**
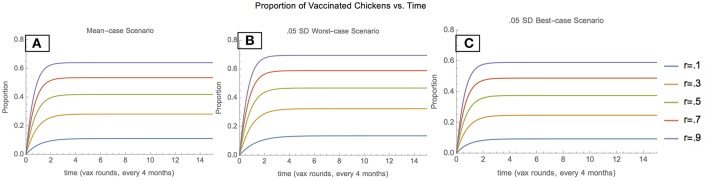
Proportion of vaccinated chickens: mean scenario **(A)**, 0.05 SD-worst scenario **(B)**, and 0.05 SD-best scenario **(C)**.

Depending on the scenario, and vaccination coverage level, the proportion of the population comprising vaccinated chickens also varies. In the mean-case scenario, the proportion of vaccinated chickens increases and then stabilizes at progressively higher values as vaccination increases, indicating that vaccination is effective in maintaining a Newcastle-immune pool of chickens (Figure 2A). In the 0.05 SD-worst-case scenario, the proportion of vaccinated chickens stabilizes at higher values than in the mean-case scenario for each vaccination coverage, indicating that when unvaccinated chickens enter the population at lower rates and leave at higher rates, vaccination plays a greater role in maintaining the chicken population (Figure 2B). In the 0.05 SD-best-case scenario, the proportion of vaccinated chickens stabilizes at lower values than in the mean case for each vaccination coverage level, indicating that when unvaccinated chickens enter the population at higher rates and leave at lower rates, vaccination plays a lower role in maintaining the chicken population (Figure 2C).

### 3.2. Population Metrics

This population growth model yields predictive insights into vaccination coverage thresholds required for two standard metrics used to evaluate the potential of vaccination interventions to alter chicken population dynamics: the acquisition of herd immunity and population doubling.

The model predicts that herd immunity, defined as 85% or more of a chicken flock having Newcastle disease immunity via vaccination at each time point, is not achievable within 5 years through vaccination alone. In contrast, population doubling is achievable. The model predicts that in the mean case, population doubling is achieved over 5 years (15 vaccination campaigns), at consistent vaccination coverage levels of 37.5%.

As parameter values approach extremes, vaccination becomes insufficient to enable doubling (worst-case), or unnecessary for doubling to occur (best-case) (Table 4), hence indicating that at extreme parameter values, the population inflow and outflows impact population growth much more than vaccination.

**Table 4:** The vaccination rate needed for population doubling in 5 years is calculated for parameter values assuming different values with respect to their standard deviations.

**Case**	**Vaccination rate for population doubling within 5 years**
± 0.15 SD Worst-case	Not Possible
± 0.1 SD Worst-case	83.5%
± 0.05 SD Worst-case	61.3%
± 0.01 SD Worst-case	42.4%
**Mean case**	**37.5%**
± 0.01 SD Best-case	32.5%
± 0.05 SD Best-case	11.9%
± 0.1 SD Best-case	Vaccination not necessary
± 0.15 SD Best-case	Vaccination not necessary

*The worst-case scenarios represents the rate for parameters plus the given fraction of standard deviation for each outflow parameter (deaths, sales, eating) and minus the given fraction of standard deviation for each inflow parameter (hatches, purchases). The best-case scenarios represent the rate for parameters minus the given fraction of standard deviation for each outflow parameter and plus the given fraction of standard deviation for each inflow parameter*.

**Figure 3:**
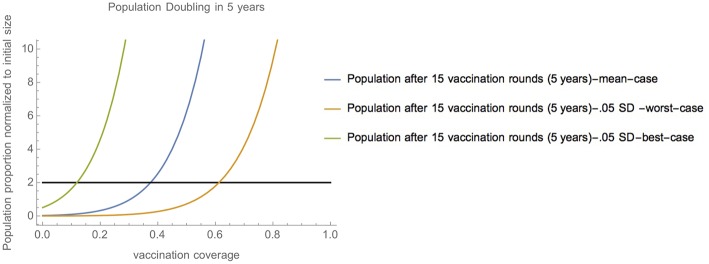
Range of vaccination coverage needed for population doubling—Vaccination coverage required to achieve population doubling in 15 vaccination campaigns (5 years) in scenarios close to the mean (mean-case scenario, 0.05 SD-worst-case scenario, and 0.05 SD-best-case scenario).

Specifically, in non-extreme cases close to the mean (0.05 SD-worst-case scenario, and 0.05 SD-best-case scenario) vaccination coverage between 11.9% and 61.3% enable population doubling over 5 years (15 vaccination campaigns) (Figure 3). Figure S1 shows how these values are correspondingly higher in the event that vaccination is imperfectly effective. Notably, population doubling within 5 years (15 vaccination campaigns) is still possible except in the 50% effectiveness, 0.05 SD-worst-case scenario).

### 3.3. Vaccination Coverage Analysis

**Figure 4:**
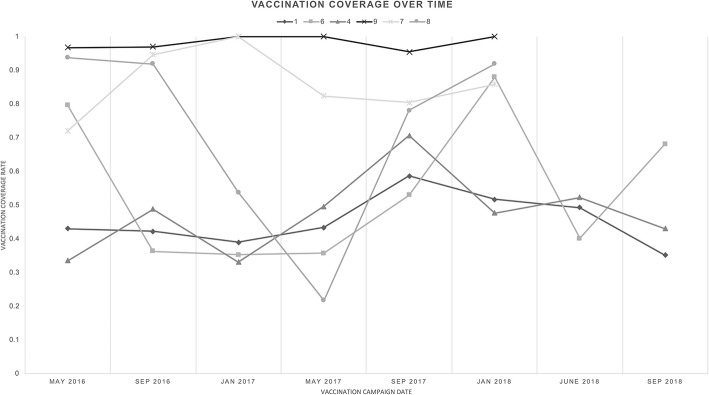
Vaccine coverage over time, across pilot communities—The community vaccination percentage at each time point for each community is shown.

Data from the initial pilot phases of the vaccination program suggest that, at present, most communities are on track to achieve population doubling over five years in the mean case (Figure 4); however, there are periodic timepoints where vaccination levels drop below the 37.5% necessary for population doubling. Overall, it seems that community vaccination levels are moderately high, and moving toward consistent achievement of the 37.5% threshold.

## 4. Discussion

Our results find that stabilization and population growth of chicken flocks are possible through moderate, consistent vaccination coverage. Population growth is critical to enabling stable, sustainable chicken flocks. A growing population can reliably provide more chicken for economic activity and consumption. Here, population doubling provides a metric for quantifying how vaccination affects population growth. The model shows that in the mean-case scenario, 37.5% vaccination coverage over 15 rounds (5 years) would enable population doubling. In worst-case scenarios, the vaccination coverage needed is higher and in extreme scenarios, no amount of vaccination success would lead to population doubling. Our model results also indicate that in best-case scenarios the vaccination coverage needed is lower and may be unnecessary in some regions according to extreme best-case scenarios of chicken productivity.

Based on this analysis, it appears that regardless of the exact population dynamics operating in chicken populations from year-to-year, consistent vaccination coverage must be prioritized. In particular, the solutions to the model assume 100% efficacy of the vaccination in conferring 4-month immunity; however, this may not be true. In the event that the vaccination is imperfectly effective, vaccination coverage will need to be higher to ensure that the effective vaccination coverage meets the population doubling target. Moreover, the model assumes that no diseases other than Newcastle disease impact chicken flocks. In reality, while we lacked data on these diseases, observations by the community vaccinators inform us that other diseases including fowl pox, scaly leg mite infection, fowl cholera, and flea infestations continue to impact chicken flocks. Therefore, vaccination coverage may need to be higher to further lower Newcastle disease related deaths, in order to compensate for other illness deaths.

While population growth is readily achievable, our results suggest that population-level herd immunity is not achievable through community vaccination alone. Attaining herd immunity is important as it provides flock-wide immunity against Newcastle disease outbreaks by preventing the spread of the disease. Prior studies have estimated that 85% of the flock must consist of vaccinated chickens at all times to achieve herd immunity against Newcastle disease (26). In our scenarios, we determined that triannual vaccination alone cannot enable the 85% herd immunity threshold. Given that vaccinated chickens continuously exit the population due to non-illness death, or may fail to be re-vaccinated, it is difficult to sustain high proportions of vaccinated chickens. Moreover, due to the large numbers of chicks that are hatched during the four-month vaccination intervals, unvaccinated chickens can remain a high proportion of the population.

However, it is important to note that immunity may still occur on a household-level scale, as individual families maintain high vaccination levels among their flock. Furthermore, during a Newcastle disease outbreak, representing an extreme worst-case scenario, unvaccinated chickens may die at such high rates that vaccinated chickens come to dominate the population, and slow the spread of disease. For these reasons, we believe that vaccination still provides substantial protective benefits during an outbreak.

Ultimately, these results demonstrate that while vaccination interventions alone may not provide herd immunity, they can play a crucial role in enabling the growth of chicken populations. Population doubling and herd immunity are metrics that provide standardized quantifications of chicken population dynamics, though with secondary utility to the given application since (1) rapid population doubling need not be a goal of vaccine interventions—rather the goal is to enable stable flocks sufficient for consumption and sales of eggs and chickens, and (2) full herd immunity may not be necessary for communities to reap the benefits of vaccination interventions. Perhaps the most important feature of these results is that moderate vaccination levels can transition chicken populations from exponential decline to growth, hence enabling surplus chicken for economic activity.

### 4.1. Vaccination Intervention

Preliminary data from the vaccination intervention suggest that vaccination coverage is generally sufficient for population stabilization and growth, but there are periodic lapses, where low vaccination coverage hinders progress.

In general, we believe there are numerous reasons why people vaccinate. Most importantly, community members are receptive to the benefits of disease protection that the vaccination provides. Furthermore, there can be social pressure to vaccinate due to the community wide benefits of Newcastle disease immunity. There are also perceived benefits on chicken growth rate that may engender owners to use the vaccine. These factors can be capitalized on to encourage vaccination.

There are also numerous reasons why community members are hesitant to vaccinate. First, the vaccine is a significant financial investment. There are also doubts as to its effectiveness, and some community members express concern about having their chickens repeatedly handled by vets and vaccinators. Others may be concerned that vaccination increases susceptibility to other diseases. Addressing these concerns at the community level is integral to the success of the vaccination intervention.

From our data, we observe that larger Malagasy communities exhibit lower vaccination coverage (Figure 4). Community 1, with 189 households, generally had vaccination coverage below 50%; however, in September 2017 the coverage spiked near 60%, which is encouraging. In contrast, Community 9, with 11 households, consistently exhibited vaccine coverage near 100%. This may indicate that high vaccination coverage levels are more readily achieved in small communities where it is simpler to reach all families and round up chickens. This could also be indicative of information spread and social pressure- in smaller communities, it is easier to inform all households of the vaccination intervention and encourage them to participate. It may be harder to attract participation of everyone in larger communities. Moreover, smaller, remote communities have less access to outside food sources, making sustainable chicken an increasingly crucial food source, attracting greater investment.

One exception to this trend was Community 8. In this relatively small community of 52 households, vaccination coverage began at higher than 90%, but dropped near 20% before recovering. Based on the account of the field team, we believe this drop was due to community members believing that the vaccination spread Fowl Pox, another concern-raising poultry disease, albeit with far lower fatality rates than Newcastle disease. After reminding vaccinators to minimize direct handling of poultry during vaccination, reminding veterinarians and vaccinators to wash hands between households, and talking with community members to emphasize the safety of the vaccine, vaccine coverage began to recover. This is an encouraging sign and emphasizes the importance of community centered interventions.

An opportunity for further improvement lies in minimizing disparities between the community-level vaccination coverage, and the average household-level vaccination coverage. Even while the community-level vaccination coverage is high, there can be pockets of households not vaccinating due to low overall vaccination adoption. Families with many chickens may be vaccinating at high levels, while a large number of families with few chickens are vaccinating at lower levels. This is undesirable, as it could allow for enclaves of unvaccinated chickens to exist, which retain susceptibility to Newcastle disease outbreaks, even as population level immunity increases.

Chicken is a particularly promising source of sustainable food in Malagasy communities for several reasons. First, for the local Malagasy people, chicken is preferred over other meats for its taste (32). Second, chickens are an acceptable source of food for nearly all sub-populations within local Malagasy culture (i.e., across religions, socio-economic status, genders, and age classes) (4, 33). While many Malagasy households maintain *fady*, or taboos, against the consumption of certain meats, there are exceedingly few against chicken consumption (34). Third, the economic dimensions of meat prices are favorable. Chicken is more expensive per kilogram than wildlife (4), yet people prefer the taste of chicken. If the productivity of chicken raising could be increased, the price may decrease. Households may benefit in a variety of ways, including increased chicken and egg consumption, and earned income from chicken sales. Fourth, in Malagasy culture, poultry are often seen as a gendered female asset (35, 36). By empowering women, this intervention may help the entire household, and can serve to protect especially vulnerable women and children (10, 37). Finally, of all the potential livestock that communities can raise adjacent to the rainforest, chickens are least likely to damage the environment (37). Unlike cattle, pigs, goats, or other mammalian livestock, chickens do not require wide land ranges, and their location can be easily controlled (10). Populations can therefore be maintained at desired levels, ensuring productivity and stability of stocks, leading to increased consumption of chicken and eggs (10, 11). Finally, the meat and eggs from free-range scavenging chickens require minimum housing and feed investment (10).

Despite the clear attractiveness of chicken, vaccination interventions will not be successful without the achievement of high and consistent vaccination coverage.

### 4.2. Policy Recommendations

Based on the results of the model, and the practical experiences of the vaccination field team, we recommend prioritizing community-centered interventions, price optimization, improving vaccinator benefits, and continuing education via the following recommendations.

#### 4.2.1. Community Centered Intervention

The vaccine intervention must maintain a community-level focus, and community stakeholders must be continually consulted and treated as equal partners. Local people have a wealth of knowledge regarding the environment in which they live and the needs of their community, as well as vast lived experience with the factors the vaccination intervention tries to address. Working in partnership with local people is not only the morally right thing to do, it also helps ensure the success of the vaccination intervention by maximizing community buy-in, and increases intervention efficacy by incorporating local knowledge.

#### 4.2.2. Pricing Options to Drive Adoption

Steps must be taken to continue making the vaccine affordable to local people. Currently, the vaccination costs Ar100 MGA (the Malagasy currency) per dose. This amounts to $0.03 USD per dose, or roughly $0.10 USD per chicken per year. While this is relatively inexpensive, vaccinating an entire chicken flock (often as many as 10–20 chickens per family) can represent a not insignificant financial investment, against a mean monthly family income of $23.17 USD or $278.04 USD annually (29). In particular, these are mean incomes, and some families necessarily see lower cash flow, making the cost burden higher. Due to the importance of even vaccination coverage across the community, it is important that the vaccine be affordable to everyone. This investment can be recouped through the sale of chickens and eggs, or in cost savings on other food purchases; however, these are long term benefits. In the short term, the cost of the vaccination may be a deterrent for some community members and innovations to reduce the cost of the vaccine, or obtain government or NGO subsidy for the cost represent avenues by which adoption can be increased. Moreover, community members may not perceive the full value of the vaccination, and hence be hesitant to participate. For these reasons, there are also opportunities to implement incentive pricing models, where families can obtain a discount for vaccinating multiple chickens or purchasing follow-up doses. Alternatively, tiered pricing could be considered, where new families joining the intervention could also receive a discount to encourage early adoption, and as a trial period. Greater consideration of financing strategies must be considered as well, as we do not consider the costs borne by IMVAVET and other groups responsible for the vaccination.

#### 4.2.3. Improving Vaccinator Benefits

In the current distribution scheme, master and community vaccinators are relied upon to deliver and administer vaccinations; however, their costs are high. They are required to travel to the location where the vaccine is stored every four months, and must find room and board for the duration of the vaccination campaign. This takes away time from their agricultural and familial duties. Since the vaccinators are such an integral part of the vaccination intervention's success, steps must be taken to make participation more attractive, and mitigate costs.

#### 4.2.4. Continuing Education

While high vaccination coverage can help prevent outbreaks of Newcastle disease from spreading, it doesn't guarantee population growth, nor does it eradicate Newcastle disease. There will continue to be incidences of Newcastle disease, and without continued vaccination maintenance, protection will be lost. Therefore, it is important that community vaccinators make clear to community members the importance of continued vaccination adherence, even as the population begins to stabilize (19). There is also a need for education on chicken husbandry, including housing, nutrition, brood hen and egg management, young chick care, and prevention and treatment of other poultry diseases. Community vaccinators should integrate education on best feeding practices for providing more nutritious feed to strengthen immunity, increase growth, and improve the success of egg laying. Biosecurity measures should also be discussed, so that communities understand how the virus is spread, how to quarantine newly purchased chickens, and how to isolate and dispose of infected chickens during potential outbreaks, all to reduce Newcastle disease transmission (13). These educational efforts are complementary to the vaccine intervention.

### 4.3. Community Impacts

If successful, vaccination interventions can result in a growing chicken population, with drastically lowered susceptibility to outbreaks of Newcastle disease. At this point, we expect that chicken flocks would be able to provide consistent, reliable food sources to remote Malagasy communities.

The stabilization of chicken flocks in Malagasy communities will have numerous positive impacts on the environment, on the economy, and on human health.

Prior studies in Myanmar, Tanzania, and South Africa have demonstrated that Newcastle disease vaccination programs can have direct impacts on communities by increasing chicken and egg consumption, enabling larger flock sizes, decreasing Newcastle disease deaths, and generating increased earned income from poultry sales (20–22, 38). Furthermore, studies of the I-2 ND vaccine (23, 39) have confirmed its efficacy in inducing Newcastle disease immunity in poultry flocks and in reducing the incidence of Newcastle disease outbreaks (40). Conventional epidemiological modeling (26) has shed light on the potential for Newcastle disease immunity to stabilize chicken flocks; However, to our knowledge, our study is unique in that it attempts to quantify the vaccination coverage levels needed to achieve population doubling and herd immunity. Future work is needed to analyze how our predictions match physical data.

Our study expands the existing body of knowledge on Newcastle disease vaccination programs by modeling the impacts of vaccination on chicken flocks undergoing realistic population dynamics and the standard triannual vaccines. This allows us to draw conclusions on the vaccination coverage levels needed to reap the benefits of a vaccination intervention. Moreover, we present a mathematical modeling framework that takes into account real data on chicken population dynamics, allowing for tailoring of a vaccination intervention to the given field setting. Finally, we present data on the early stages of a promising vaccination campaign in remote communities in Madagascar, that we hope can be expanded to provide a model for national and international campaigns in similar settings.

In Madagascar, we hope that enabling chickens to serve as a sustainable source of food will provide similar direct benefits to those reported in previous vaccination trials (20–22, 38), which can create the following indirect benefits described in the literature.

#### 4.3.1. Environmental

Reliance on domesticated meats may decrease reliance on wild meats. This leads to decreased hunting of wildlife. This is beneficial to the environment, as overhunting has led stocks to decline, and threatened the status of many species (7, 32). Quantitative work is still needed to ascertain to what extent the consumption of chicken and wildlife are inversely related; however, research has found that chicken is consistently the first or second top taste preference among local people, only occasionally behind the lemur species *Varecia variegata* (32). Given this finding, we suspect that chicken consumption will replace a large portion of wild meat consumption. This can help reduce overhunting, hence contributing to the preservation of biodiversity in Madagascar.

#### 4.3.2. Economic

Surplus eggs and chickens can be sold by community members to provide increased income. This income can be used to fund beneficial activities such as healthcare and education, or to purchase other foods (10, 11).

#### 4.3.3. Community Health

Communities will see improved health due to increased protein consumption from chicken and eggs (41). Currently, in the Malagasy communities we study, eggs are the least frequently consumed food group (being eaten on only 3.1% of days) (42). The increased sustainability of chicken flocks and resulting egg availability can change this, allowing communities to reap the nutritional benefits of egg consumption. Food security and nutrient availability is also increased due to the consistent food source provided by domesticated poultry (20). Moreover, families who sell surplus chicken and eggs gain additional income with which to purchase additional diverse food sources, such as beef, pork, and other market foods. Many of these foods provide nutrients not typically available to Malagasy families due to their expense. Since chickens command a substantial market price, and are in high demand, their sale will enable the purchase of these other foods. In addition to dietary improvements, reliance on domesticated poultry rather than wildlife decreases the risk of zoonotic disease transmission (43).

## Data Availability Statement

The datasets generated and analyzed for this study can be found in the Harvard Dataverse at https://doi.org/10.7910/DVN/477XH9 (44). Data collection is still in progress; please contact the authors for the most updated version of the database.

## Ethics Statement

Data concerning chicken population dynamics, trade, sale, and husbandry conditions were deemed not to require an IRB by the Harvard TH Chan School of Public Health's Office of Human Research Administration's Protocol IRB13-1862. Data concerning human dietary practices have been de-identified according to our approved research protocol, including verbal informed consent for all research participants (HSPH OHRA Protocol 22826). Written informed consent was deemed culturally inappropriate in this region where many people are illiterate and signing contracts was not common practice. Records of oral consent were maintained on our study roster sheets. All consent procedures were approved by the IRB at the Harvard TH Chan School of Public Health's Office of Human Research Administration (Protocol 22826).

## Author Contributions

CG and GC conceptualized the poultry vaccination intervention. AA, SI, and CG designed the research and analysis. CB, MR, CG, MH, EA, and HR conducted field studies and data collection. CB and CG administered the project. AA and SI performed the modeling and analysis. GC, CB, and CG acquired funding. AA prepared the original manuscript. All authors reviewed and edited the manuscript.

### Conflict of Interest

The authors declare that the research was conducted in the absence of any commercial or financial relationships that could be construed as a potential conflict of interest.
