# Supplementary figures and images for: Modeling the Impact of Newcastle Disease Virus Vaccinations on Chicken Production Systems in Northeastern Madagascar
Source: Front Vet Sci. 2019 Sep 26;6:305. doi: 10.3389/fvets.2019.00305 (PMC6775217; doi:10.3389/fvets.2019.00305)

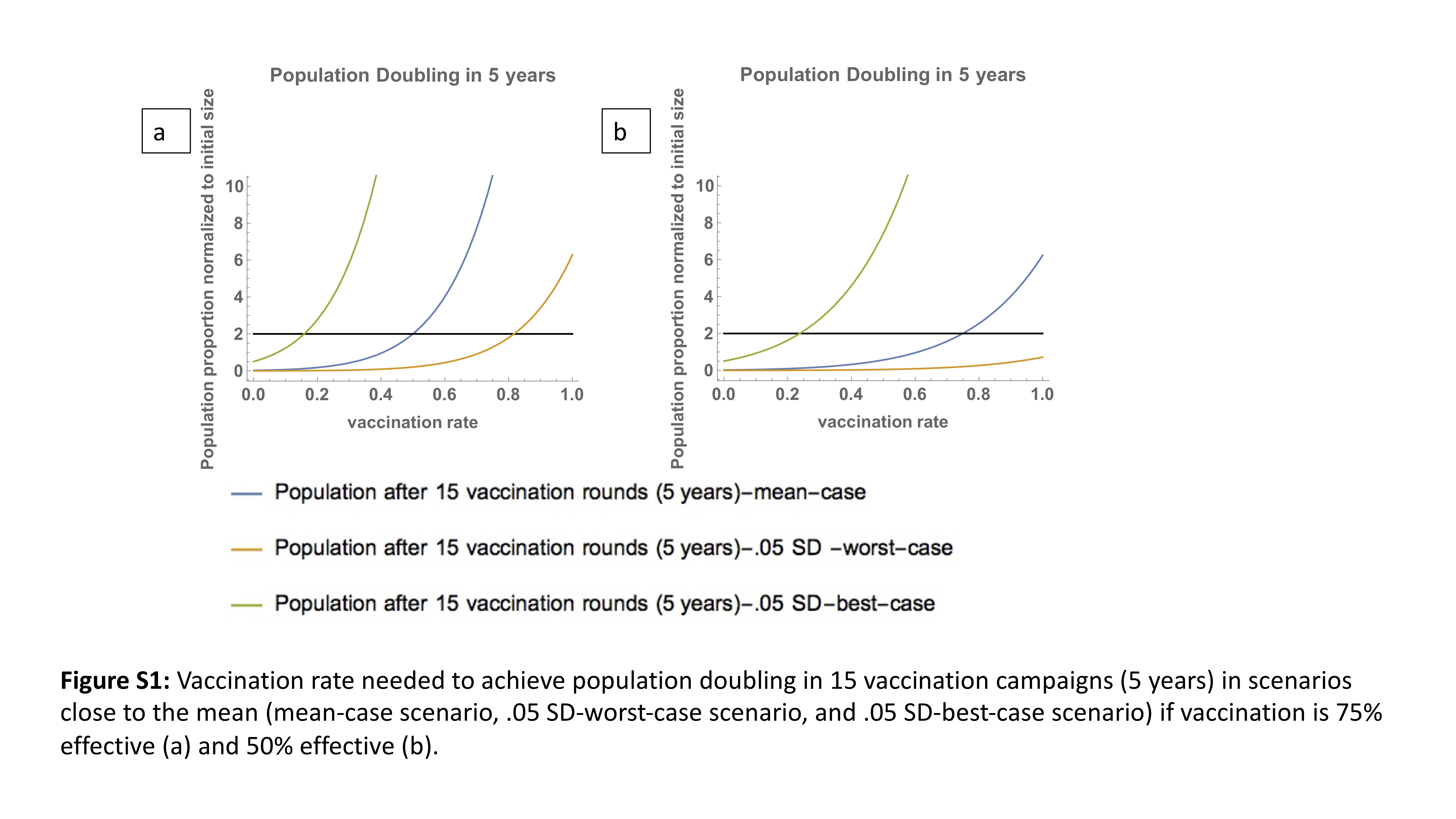

Supplement: Supplementary file 1 [file Image_1.tiff]
